# Virtual Reality–Based Neurorehabilitation Support Tool for People With Cognitive Impairments Resulting From an Acquired Brain Injury: Usability and Feasibility Study

**DOI:** 10.2196/50538

**Published:** 2024-03-18

**Authors:** Alba Prats-Bisbe, Jaume López-Carballo, Alberto García-Molina, David Leno-Colorado, Alejandro García-Rudolph, Eloy Opisso, Raimon Jané

**Affiliations:** 1 Institut Guttmann Institut Universitari de Neurorehabilitació adscrit a la Universitat Autònoma de Barcelona Badalona Spain; 2 Universitat Autònoma de Barcelona Bellaterra Spain; 3 Fundació Institut d’Investigació en Ciències de la Salut Germans Trias i Pujol Badalona Spain; 4 Department of Automatic Control Universitat Politècnica de Catalunya (UPC)-BarcelonaTech Barcelona Spain; 5 Centro de Estudios en Neurociencia Humana y Neuropsicología Facultad de Psicología Universidad Diego Portales Santiago de Chile Chile; 6 Institute for Bioengineering of Catalonia (IBEC) The Barcelona Institute of Science and Technology Barcelona Spain; 7 Biomedical Research Networking Center in Bioengineering Biomaterials and Nanomedicine (CIBER-BBN) Barcelona Spain

**Keywords:** acquired brain injury, virtual reality, head-mounted display, neurorehabilitation, usability, feasibility, co-design, multidisciplinary experiences, immersive serious games

## Abstract

**Background:**

Acquired brain injury (ABI) is a prominent cause of disability globally, with virtual reality (VR) emerging as a promising aid in neurorehabilitation. Nonetheless, the diversity among VR interventions can result in inconsistent outcomes and pose challenges in determining efficacy. Recent reviews offer best practice recommendations for designing and implementing therapeutic VR interventions to evaluate the acceptance of fully immersive VR interventions.

**Objective:**

This study aims to evaluate the usability and feasibility of a co-designed VR-based neurorehabilitation support tool by conducting multiple proof-of-concept trials in a sample of patients with ABI within a hospital setting.

**Methods:**

A single session deploying custom immersive serious games to train cognitive functions using a new-generation head-mounted display was conducted among a sample of inpatients with ABI. Structured questionnaires were administered at the end of the session to evaluate the usability of the system and the intervention, participants’ familiarity with the technology, and any adverse effects related to cybersickness. Additionally, the training duration while wearing the headset and the demographic characteristics of the participants were considered.

**Results:**

A total of 20 patients with ABI participated in a 1-hour proof-of-concept trial. The mean usability score was 37 (SD 2.6) out of 40, the technology familiarity level was 9.2 (SD 2.9) out of 12, and the Simulator Sickness Questionnaire total score was 1.3 (SD 2). On average, participants wore the headset for approximately 25.6 (SD 4.7) minutes during the intervention. There were no substantial differences in usability and technology familiarity levels based on patients’ etiology or age, with no notable symptoms of cybersickness reported. Significantly strong correlations were noted between cybersickness symptoms and various usability categories, including exposure, motivation, interactivity, task specificity, and immersion aspects. Further, there was a significant association between the intervention time and the number of tasks performed (*P*<.001). Furthermore, patients who derived enjoyment from VR sessions expressed a heightened interest in incorporating VR into their daily neurorehabilitation practice (*P*<.001). Moreover, oculomotor issues were found to be highly sensitive to the onset of disorientation sickness symptoms (*P*<.001).

**Conclusions:**

Through a collaborative approach, this study showcases the usability and feasibility of a VR-based support tool for cognitive rehabilitation among inpatients with ABI. Key components of such interventions encompass a multidisciplinary array of immersive experiences integrating neurorehabilitation principles and serious games techniques.

## Introduction

### Background

Acquired brain injury (ABI) is any postnatal brain damage that is not hereditary, congenital, or degenerative [1], and encapsulates 2 main categories, namely, traumatic brain injury (TBI) and non-TBI [2]. TBI is an external traumatic event in which injury to the brain is sustained. It is the most frequent etiology of ABI and is primarily caused by falls and road injuries. In 2016, there were 27.08 million new cases of TBI and 55.5 million prevalent cases worldwide [3]. The incidence of TBI is likely to continue rising, driven by factors such as population growth, aging demographics, and increased motor vehicle usage. By contrast, non-TBI arises from internal disease processes, such as brain tumors, causing damage to brain tissue. The primary cause of non-TBI is stroke, with ischemic stroke accounting for 62.4% of all new strokes globally, followed by hemorrhagic stroke at 37.6% [4]. In recent years, there has been a significant increase in stroke rates among young individuals, a trend expected to persist, especially in low-income countries. ABI not only results in health deterioration and disability for affected individuals and their families but also imposes a substantial burden on health care systems and economies due to lost productivity and high health care costs [2].

Individuals with ABI exhibit adverse outcomes across multiple functional domains, encompassing sensorimotor, cognitive, and behavioral areas, which impede the performance of basic activities of daily living [1]. Regarding cognitive function, deficits commonly manifest in attention, memory, and executive functions [4]. The majority of patients with TBI experience challenges with sustained, selective, or divided attention, along with diminished information processing speed. Memory issues often involve a heightened rate of forgetting, as well as slower, disorganized, and incoherent learning compared with individuals without TBI. Additionally, patients with TBI commonly exhibit executive function alterations, including difficulties in planning, limited mental flexibility, reduced inhibitory ability, and challenges in verbally recalling phonetic categories [5,6]. Cognitive impairment following a stroke varies based on factors such as the nature of the stroke, the specific brain regions affected, and the stage of recovery. Individuals may exhibit hemispatial neglect as well as various types of visuoperceptive and visuospatial impairments. Additionally, deficits in verbal memory and language-related issues are common, including aphasia, which can affect writing and reading abilities [6,7].

Although some impairments may show improvement over time, recovery rates vary as a result of differences in the baseline characteristics of individuals [6]. Furthermore, despite the distinct disease processes and medical issues associated with TBI and non-TBI, patients often receive treatment and rehabilitation in the same hospital facilities. To achieve optimal clinical outcomes for all patients with ABI, health care professionals need to deliver personalized and targeted treatments, necessitating a comprehensive understanding of the pathology across different categories of ABI [2].

Neurorehabilitation is a meticulously supervised process designed to assist individuals with ABIs in reclaiming their functional abilities and enhancing their quality of life. Fundamental components of neurorehabilitation encompass a spectrum of expert and multidisciplinary assessments, the implementation of realistic and goal-oriented tasks, and the evaluation of clinically appropriate outcome measures. Importantly, this evaluation also takes into account the perspectives of both the patient and their family [8]. Neurorehabilitation services serve as a bridge between isolation and exclusion, often representing the initial stride toward attaining fundamental rights. Health, indeed, is a fundamental right, and neurorehabilitation stands as a potent service that fosters personal empowerment, enhances independence, and notably facilitates the return to work and active participation within the community [1,8,9].

Virtual reality (VR) is emerging as a swiftly advancing technology, garnering recent popularity as a promising support tool for neurorehabilitation among individuals with ABI [10-13]. Using VR in rehabilitation represents a versatile, captivating, and multifaceted approach capable of addressing patients’ sensorimotor and cognitive capacities, thereby eliciting positive responses. It enhances treatment compliance while augmenting levels of functioning and overall quality of life [14]. VR provides a platform to simulate real-life scenarios and ecologically valid activities within a safe and controlled environment [15].

As the term “virtual reality” can encompass various computer-based rehabilitation system types across studies and may influence the feasibility and efficacy of interventions, maintaining consistent terminology is crucial [12,16]. In 1999, Brooks [17] defined a VR experience as “any in which the user is effectively immersed in a responsive virtual world. This implies user dynamic control of viewpoint.” Thus, for a system to be considered VR based, it must fulfill 3 conditions: it should be immersive, interactive, and true to reality.

Modern high-end VR systems can provide users with an immersive experience, wherein they feel surrounded by a computer-generated world that responds naturally and convincingly, while also minimizing side effects such as cybersickness [18]. The utilization of new-generation head-mounted displays (HMDs) enables stereoscopic perception and perspective changes based on the user’s viewpoint. Additionally, incorporating haptic controllers and precise tracking of 6 degrees of freedom allow the system to accurately recognize users’ motion (both position and orientation) in 3-dimensional space. Furthermore, contemporary computing techniques and advanced rendering methods facilitate the development of highly detailed graphics and real-time responses [19]. Consequently, users can engage in a realistic virtual environment, interacting with intuitive gestures that mimic their real-world movements. This immersive experience often leads to a profound sense of presence and may even induce a phenomenon referred to as “virtual embodiment” [11,20].

Despite the increasing interest in the utilization of VR technology, there remains a considerable degree of heterogeneity among health applications. The majority of studies using VR for rehabilitation have focused on addressing motor impairments following a stroke, rather than exploring other rehabilitation objectives or types of brain injuries [10,12]. Furthermore, it is noteworthy that the most commonly used output devices are flat screens and older-generation headsets [16]. Since the introduction of the first high-end fully immersive VR-based system commercially available in 2016 (ie, Oculus Rift [21]; Oculus VR), only a handful of studies have provided robust evidence regarding the feasibility and efficacy of new-generation immersive devices in rehabilitation [22-24]. Most reviews have indicated that the limited evidence stems not from negative or inconclusive outcomes, but from a deficiency in methodological designs that yield high-quality evidence levels [16,25]. As a result, determining whether the benefits of VR-based interventions are clinically significant remains challenging [26]. Therefore, VR-based interventions are still in the early stages of full implementation within real hospital settings. Establishing a standard operating procedure would prove beneficial for enhancing reproducibility, facilitating comparison, and promoting the generalization of findings across studies.

Recent recommendations regarding the utilization of VR-based interventions for clinical applications emphasize the significance of implementing a phased approach design for new programs, which includes conducting pilot studies to assess usability [27,28]. The customization of tasks to cater to the specific needs of individuals, along with the integration of serious gaming techniques [29], represents key advantages of VR in promoting effective neurorehabilitation [30-32]. Serious games techniques encompass various strategies such as adjusting the intensity and complexity of tasks, integrating multisensory feedback, using avatar representations, reinforcing actions with sound effects, and rewards. These techniques aim to foster a high level of engagement and sustain individual focus and motivation during rehabilitation sessions [33]. Moreover, they contribute to enhancing neuroplasticity through repetitive training, as highlighted by research studies [18,34,35].

The most recent studies on VR interventions for cognitive rehabilitation following ABI have focused on conducting detailed design and prototype evaluations of self-developed systems [36,37]. These studies underscore the significance of integrating expertise from cross-disciplinary perspectives, which has resulted in high levels of user satisfaction and low levels of simulator sickness. Additionally, the authors conducted second-phase trials to effectively evaluate the feasibility and preliminary efficacy of the VR-based intervention. Their primary findings suggest improvements in outcome measures of cognitive functions when the intervention is tailored to address the specific cognitive function, incorporating serious games techniques, using a patient-centered design approach, and administering sessions lasting approximately 30 minutes each [38-41].

### Objectives

This study aims to address the aforementioned recommendations by prioritizing the early engagement of both patients and clinicians in the development process. The approach involved the co-design of a new VR-based cognitive rehabilitation support tool, which underwent iterative system testing to elicit requirements and establish its utility, safety, and viability before progressing to large-scale studies. The co-design process included active participation from end users and a range of health professionals, including physical medicine and rehabilitation physicians, neuropsychologists, occupational therapists, physiotherapists, as well as researchers and technologists. The objective was to ensure the usability and feasibility of a fully immersive VR-based cognitive rehabilitation support tool among individuals with ABI through a multiple proof-of-concept study. This insight was crucial for formalizing the specific requirements for integrating VR into the daily practice of real hospital settings. The findings from this study may serve as a road map for developing new VR tools in this field and lay the groundwork for future high-quality studies. These studies are essential to ascertain the real efficacy and cost-effectiveness of VR-based interventions in clinical practice.

## Methods

### Overview

The methodology of this study comprised 2 main parts. First, the design and development of a VR-based cognitive rehabilitation support tool, which followed a thorough and iterative approach involving a multidisciplinary team from the Institut Guttmann, a specialized neurorehabilitation health care center. Second, patients with ABI were recruited to participate in a single session using the VR-based system within the real hospital setting, aimed at assessing the usability and feasibility of the proposed intervention.

### Study Design

In the first part, the need for acquiring a VR-based support tool was identified through interviews conducted with clinical professionals involved in the neurorehabilitation process (for detailed information, refer to Table S1 in Multimedia Appendix 1). Subsequently, a multidisciplinary team brainstormed new ideas for VR-based interventions and suggested the development of a novel cognitive rehabilitation support tool. The acquisition of a modern VR headset was planned, and strategic placement was arranged within the hospital configuration to facilitate its use. Researchers, neuropsychologists, and technologists commenced work on a phased co-design and prototyping of VR tasks targeting specific cognitive functions. These prototypes underwent testing in close consultation with the multidisciplinary team and patients with ABI. Feedback was collected, and corresponding changes were implemented for each task iteratively until maximum safety and desired functionality were ensured.

**Figure 1:**
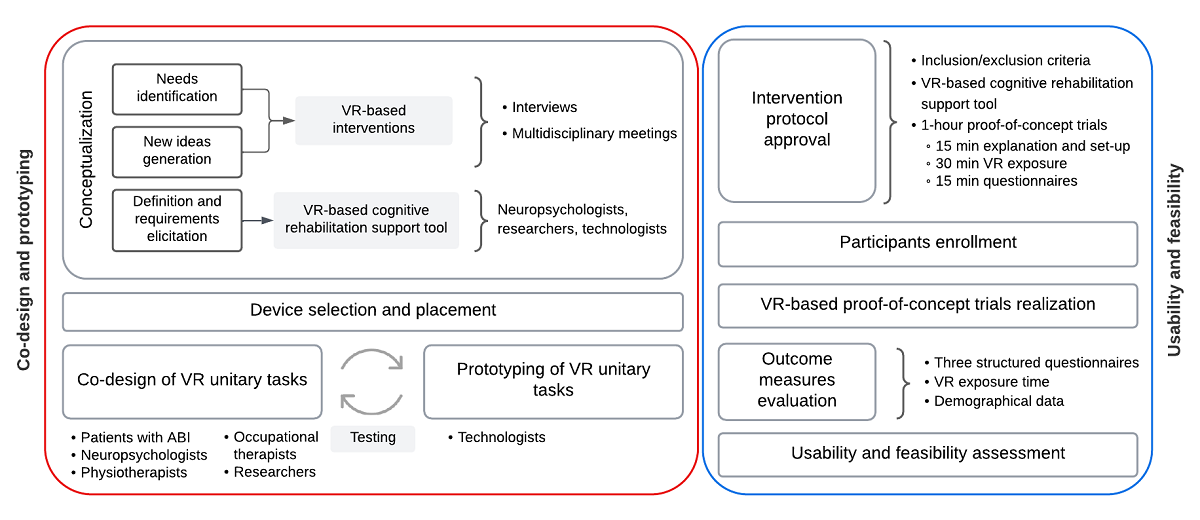
Study design methodology description, divided into 2 main parts: the co-design and prototyping phase and the usability and feasibility phase. ABI: acquired brain injury; VR: virtual reality.

The second part involved conducting a multiple proof-of-concept study to evaluate the usability and feasibility of the self-developed VR-based cognitive rehabilitation support tool in patients with ABI (Figure 1). Participants were recruited from the Institut Guttmann.

### Ethical Approval

Ethical approval for this trial was obtained from the Ethical Research Committee (CEIm) of the Fundació Unió Catalana d’Hospitals (reference number CEI 22/34), and the study was conducted in compliance with the principles outlined in the Declaration of Helsinki. Written informed consent forms were completed by all participants.

### Participants

Various profiles participated in the co-design and prototyping phase (refer to Table S2 in Multimedia Appendix 1). The initial cross-disciplinary team comprised 9 research members from the Institut Guttmann, including 3 neuropsychologists, 2 physiotherapists, 2 technologists, and 2 researchers in the field of technological innovation applied to health. Together, they developed the initial approach for the VR-based tool.

After the initial prototypes were developed and tested by the research team, additional clinical professionals, including physiotherapists, occupational therapists, and neuropsychologists, were invited to test advanced prototypes. They were asked to provide feedback as they familiarized themselves with manipulating the tool.

The most advanced prototypes, which met acceptable safety levels based on clinical criteria, were tested by 9 patients of varying ages and sexes, spanning from childhood to youth to advanced age, and with different etiologies including TBI, stroke, or brain tumor. These patients were undergoing functional training at the rehabilitation gym of the Institut Guttmann. They were required to understand basic instructions, possess sufficient mobility to manipulate a controller with at least one hand, not have epilepsy or vertigo, and be capable of wearing glasses if needed. Positive feedback was appreciated, and valuable comments and observations were collected to inform the final acquisition of the VR-based cognitive rehabilitation tool.

For the usability and feasibility assessment, all individuals admitted to the Institut Guttmann between June and August 2022 were considered for participation in the multiple proof-of-concept study if they met the following criteria:

- Presence of an ABI (moderate to severe TBI, stroke, or brain tumor).
- Age equal to or greater than 16 years.
- Presence of cognitive impairment assessed using a neuropsychological test battery.
- Well-oriented in the 3 different spheres (person, space, and time) and understands basic instructions.
- Had enough mobility to manipulate a controller with at least one hand and press any button.
- Received cognitive rehabilitation training through a not-immersive computer-generated tool named Guttmann NeuroPersonalTrainer [42].

The exclusion criteria were as follows:

- Presents linguistic (aphasia) or visuoperceptive alterations that could affect the administration and validity of the results obtained in the neuropsychological assessment battery or VR session performance.
- Psychiatric or neurological history before ABI.
- Have epilepsy or disorders associated with motion sickness.
- Patients with skull shape abnormalities who cannot comfortably hold the VR headset.

During the recruitment period, a total of 20 inpatients (9 female) met the inclusion criteria and were enrolled in the study. Among them, 7 patients had a TBI, 12 had a stroke, and 1 presented with a brain tumor.

### VR System

#### Device and Development Tools

The VR system must possess the capability to capture user actions through motor interfaces. These actions will be interpreted as requests to modify the virtual environment and sensory reactions will be transferred to the sensory interfaces. Furthermore, specific hardware capabilities, including the type of display screen, resolution, image refresh rate, and field of view, along with software attributes such as ergonomic interactions and navigation, are crucial for mitigating VR-induced symptoms and effects [43,44]. The minimal technical specifications for such a system are listed in Table 1.

**Table 1:** VR^a^ device minimal technical requirements specification.

Object	Requirement
Display screen	OLED^b^ or LCD^c^
Screen resolution	>960 × 1080 pixels per eye
Refresh rate	≥75 Hz
Field of view	≥110° diagonal
Audio	Integrated and adjustable
Sensors	6-DoF^d^ tracking, accelerometer, gyroscope, proximity, and haptic
Ergonomics	Adjustable eye comfort setting (IPD^e^); head strap
Tracked area	Up to 2 m × 2 m
Controllers	Minimum of 2 with buttons and 6 DoF

^a^VR: virtual reality.

^b^OLED: organic light emitting diode.

^c^LCD: liquid crystal display.

^d^DoF: degrees of freedom.

^e^IPD: interpupillary distance.

The HTC VIVE Pro Eye (HTC Corporation) [45], a new-generation high-end HMD and handheld controller, was selected and integrated into the hospital configuration. This device is currently commercially available in most countries and is compatible with industry-standard interfaces such as SteamVR (Valve Corporation) [46] and OpenVR (Valve Corporation) [47]. With the Unity3D (Unity Technologies) game engine [48], our team successfully created immersive, interactive, and true-to-reality virtual environments. These environments can be executed on any VR station that meets the aforementioned minimal technical requirements.

#### From Prototyping to Immersive Serious Games

A co-design approach was undertaken involving health professionals, researchers, and technologists. The multidisciplinary team engaged in discussions regarding the configuration of the VR session, addressing aspects such as duration, the number of tasks, task characteristics, and measurable data. Recognizing that individuals with ABI may have disabilities across multiple areas of functionality, the team emphasized the importance of developing a set of unitary tasks. This approach would allow for targeting different cognitive abilities and obtaining relevant outcomes separately, thereby ensuring comprehensive training for the patient.

Unitary tasks should be designed to be achievable, with clear objectives, and customized based on each patient’s specific needs to accommodate any physical or cognitive limitations they may have (eg, muscle rigidity or hypersensitivity). Participants could use 1 or 2 handheld controllers, and interactions were simplified by programming multiple buttons to perform the same action.

Tasks could be completed in either sitting or standing positions; however, to minimize the risk of falling, as reported in a previous study [49], all participants underwent the VR session while seated. Accelerations or decelerations were avoided and substituted with uniform linear motion or teleporting methods to ensure a safe and comfortable experience for the participants. This approach reduces motion sickness by requiring users to actively control their viewpoints and be responsible for initiating movement [18]. Virtual scenes were designed to be as realistic as possible, corresponding to the stimulus type (eg, a sports center for football stimuli), and the stimuli appeared within the user’s field of view. All exercises followed a dual-task approach, incorporating both cognitive and motor cues (eg, reaching visuospatial stimuli), to provide a comprehensive rehabilitation experience.

The final prototypes were attained through continuous testing and evaluations involving end users and clinical professionals. Key topics and features that underwent extensive discussion and redesign were game mechanics, interactivity, sound effects, graphic design, and variable thresholds to delineate difficulty levels. Seven immersive experiences were developed, addressing 3 different cognitive functions: attention (n=4), memory (n=1), and executive functions (n=2).

Prototyping these experiences as serious games facilitated the incorporation of appropriate feedback, including visual (V), auditory (A), and haptic (H) cueing. This approach enabled the provision of instructions, rewarding or annoying stimuli to guide users in expected motion realization, and the ability to display or perceive real-time performance results [50]. The emission of slight vibrations when interacting with a virtual object can induce the sense of having touched it. Additionally, task difficulty was adjusted to fit the patient’s therapeutic window, allowing the professional to select 1 of 3 possible difficulty levels. Each task automatically modified certain dependent variables based on the chosen difficulty level.

**Figure 2:**
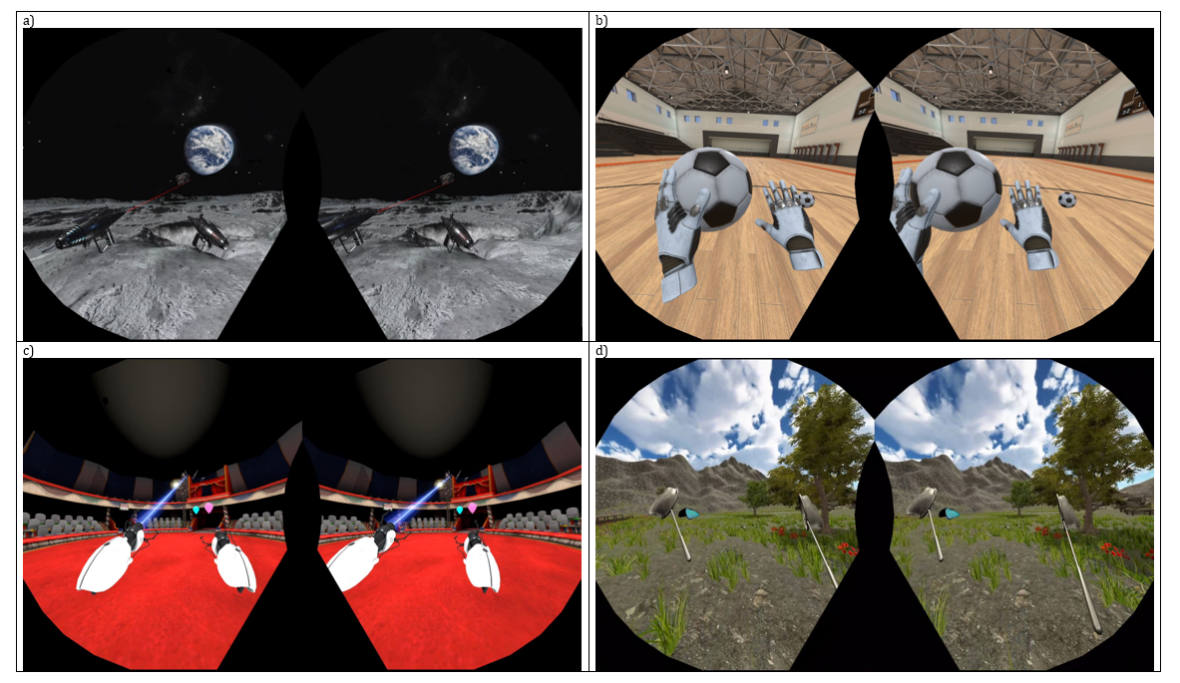
The 4 attentional immersive serious games: (A) Moon, (B) Goalkeeper, (C) Circus-I, and (D) Butterflies.

Attentional serious games consist of 4 visuospatial tasks (Figure 2). Each task involves a different presentation-interaction approach: (1) stimuli moving at different constant speeds from right to left in a straight line and then reversing direction at different heights. The user, who is stationary, must shoot them; (2) stimuli moving toward the user in a parabolic arch trajectory from different positions. The user must intercept them; (3) stationary stimuli distributed at various points within the user’s field of view. The user must shoot them; and (4) stationary stimuli appearing near the user’s left or right side while they are virtually moving forward at a constant low speed. This creates the perception that the user is moving toward the stimuli and can reach them with their hands.

**Figure 3:**
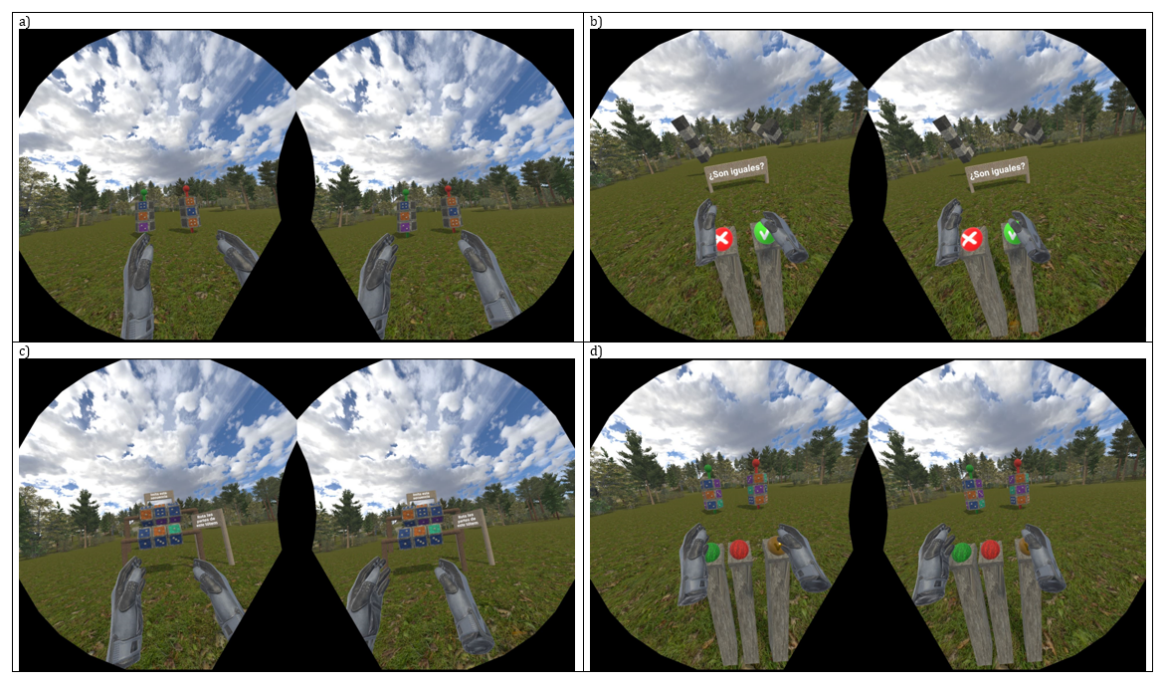
A memory immersive serious game (Totem) and its phases: (A) encoding, (B) min-interference, (C) max-interference, and (D) decoding.

One memory task was developed to train short-term and working memory within an immersive 3D naturalistic environment (Figure 3). Users could focus on the exercise they had to carry out without any external distractions. The task comprises 3 phases: an encoding phase, an interference phase (which can be configured as maximum or minimum interference), and a decoding phase.

**Figure 4:**
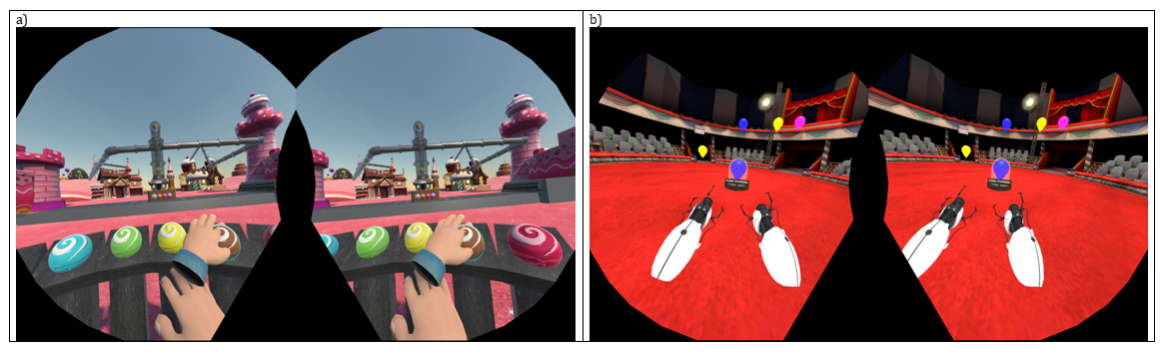
The 2 executive functions immersive serious games: (A) Conveyor-belt and (B) Circus-II.

The executive function tasks aim to train high-level cognitive abilities, such as planning, problem-solving, and decision-making. For this research, 2 tasks were developed wherein the participant is immersed in performing a repetitive task that varies in the principal instruction that must be carried out (Figure 4). The first task follows the design of a sequence imitation task, while the second exercise was designed to control automatic responses using attention and reasoning through an inhibitory control task.

During VR sessions, in-game measures were collected, including time stamps, hits/failure scores, reaction times, user-system interactions, gaze/position tracking data, and stimuli data. At this stage, an easy-to-use system with a quick set up for sessions involving a set of VR experiences addressing cognitive functions was achieved.

### Intervention

Immersive serious games were deployed on the HTC VIVE Pro Eye device, which was equipped with 2 room tracking units (infrared cameras) and 2 controllers. Once the doctor identified a potential participant, he/she or a tutor was invited to participate in the study. Enrolled patients substituted 1 hour of their cognitive treatment with traditional cognitive rehabilitation therapy with 1 hour of intervention using the VR-based system tool. All sessions were conducted between June and August 2022.

During the initial 15 minutes, the participant completed the informed consent forms and was seated in a chair or positioned in their wheelchair in a designated area within the VR system’s tracking zone. To ensure safety, clear space within the room was maintained, keeping the participant at a distance from any objects or individuals to prevent collisions. Subsequently, the VR headset and controllers were placed on the participant. The treatment provider configured the VR session via a host computer by selecting the difficulty level for each cognitive category (hard, medium, or easy) and specifying the hands involved (see photos of the set up in Multimedia Appendix 2).

The session consisted of completing various tasks, with each task lasting 4-6 minutes. The total intervention time wearing the headset was approximately 30 minutes unless the patient requested to conclude earlier. The intervention time was calculated as the sum of the duration of each task carried out, excluding the time elapsed between tasks when the treatment provider ensured that the task objectives were understood and instructed the patient on how to interact with the environment. The number of total tasks performed was also counted. When there were 15 minutes remaining until the end of the VR session, the HMD was removed, and questionnaires were administered to participants to assess their overall user experience.

### Outcome Measures

To assess the usability and feasibility of the VR-based support tool for cognitive rehabilitation in patients with ABI, 3 structured questionnaires were used (Table S1 in Multimedia Appendix 3). Additionally, information regarding the optimal dose of treatment and patients’ age, based on the duration of time spent performing VR tasks, along with demographic data, was collected.

The first questionnaire comprised a 5-point Likert scale, ranging from “5=fully agree” to “1=fully disagree,” assessing system usability and acceptance based on the participant’s perception. The responses were related to the sense of presence, dimensions matching, the ability to see and differentiate objects, interactivity, task specificity, task difficulty, motivation, enjoyment, and errors. Following this, 3 questions were posed regarding the frequency (on a 5-point Likert scale, ranging from “5=all the time” to “1=never”) of using various new technologies to gauge the familiarity level. Finally, the Simulator Sickness Questionnaire (SSQ) [51] was used to evaluate side effects by measuring users’ level of sickness symptoms such as nausea (N), oculomotor problems (O), and disorientation (D). Each of the 16 items in the SSQ is rated on a 4-point scale: 0 (none), 1 (slight), 2 (moderate), and 3 (severe). Participants were instructed to indicate the severity of each symptom they experienced during or after the VR exposure by selecting the appropriate rating for each item.

For the Usability Questionnaire (UQ) and the Technology Familiarity Questionnaire (TFQ), the value for the “worst” condition answer will count as 0, and the value for the “best” condition answer will count as 4. As the UQ has 10 questions, the maximum total score can be 40. A higher usability score indicates that the system is more useful and feasible for implementation in a hospital setting for patients with ABI during neurorehabilitation. The maximum total score for the 3-question TFQ can be 12, indicating a greater acceptance of new technologies.

By contrast, the total score for the SSQ can range from 0 to 48, with significant symptoms indicated by scores between 10 and 15, concern for scores between 15 and 20, and scores over 20 indicating a problem with the simulator. Their usage permitted detailed analysis of the distribution of nausea, oculomotor, and disorientation symptoms elicited by the experimental manipulation. If any score falls within a concerning range, it should be studied separately because this scale was originally designed for military flight simulators and may appear overly strict when applied to nonaviators [52]. However, this questionnaire is one of the most widely used ones for assessing cybersickness in immersive VR rehabilitation [53]. Thus, its use allowed for comparison with previous research.

Structured questionnaires containing numbered questions, accompanied by keywords pertaining to usability, technical familiarity, and side effects, along with the complete question sentences, are available in Table S1 in Multimedia Appendix 3.

### Statistical Analysis

We aimed to recruit enough inpatients with ABI to identify all usability problems in the design [54] and the early stage of this self-developed VR tool and to gather sufficient data to estimate the SD of measured outcomes for planning a subsequent larger trial [55]. Recent studies, which involved new-generation headsets, customized VR-based rehabilitation tools, focused on patient needs, tested the system in samples ranging from 11 to 35 patients with ABI, and found that VR was accepted and feasible for rehabilitation [37,38,56].

Descriptive analyses were conducted to establish recruitment, acceptance, and completeness, using demographic information, questionnaire scores, measures of intervention duration, and the number of tasks completed. Descriptive statistics data from participants with TBI and stroke were reported separately. As only 1 participant had a brain tumor, their data were not included in the etiology-group comparison. However, their data were included in the age-group comparison established for future eligibility criteria.

The calculations were conducted using Microsoft Excel. The R package (R Foundation) *corrplot* [57] was used to graphically represent the scores obtained in the questionnaires and compare them according to age and etiology. Additionally, the same package was used to explore the correlation matrix between SSQ subscale symptoms, usability categories, technology familiarity, in-game measures, and some demographics. *P* values with a significance level <.05 and correlation coefficients (*r*, ranging between –1 and +1) were provided to aid in determining the statistical significance and the direction and intensity of correlations.

## Results

### Sample Characteristics

A total of 20 inpatients with ABI participated in this usability and feasibility study. The sample mean age was 38.3 (SD 14.1) years, with a mean time since injury (TSI) of 4.7 (SD 1.5) months. The total scores obtained for each of the 3 questionnaires administered (ie, UQ, TFQ, and SSQ) were 37 (SD 2.6), 9.2 (SD 2.9), and 1.3 (SD 2), respectively. Finally, the total mean duration of each intervention across all participants was approximately 25.6 (SD 4.7) minutes, while the number of tasks completed was 5.1 (SD 1).

Among the 7 patients with TBI, 4 reported a severe level of impairment according to the Glasgow Coma Scale (between 3 and 8) [58]. Among the 12 patients with stroke, 7 had ischemic strokes and 4 had hemorrhagic strokes. There were 2 cases of minor stroke according to the National Institute of Health Stroke Score (NIHSS; ranging from 0 to 42: 0, no deficit; minor impairment, 1-4; moderate, 5-15; moderate to severe, 16-20; and severe impairment 21-42) [59]. Seven patients had moderate stroke severity, and 2 presented with moderate to severe stroke. The patient who had a brain tumor underwent surgery for resection of a pituitary macroadenoma.

Patients underwent a battery of neuropsychological tests before being incorporated into the study; 8 of them had alterations in the cognitive function of attention, 8 presented with memory impairment, and 18 had difficulty performing executive functions. Five patients had completed advanced studies (>12 years of schooling), while 8 had an intermediate level of education (between 8 and 12 years of schooling) and 6 completed primary education (<8 years of schooling).

Moreover, one patient presented with hemispatial neglect, 6 had left-side hemiplegia, and 4 had visual-field defects, including homonymous hemianopia, diplopia, or limited gaze. The individual demographics and some clinical data are reported in Table 2. For more details and complete information, please refer to Table S1 in Multimedia Appendix 4.

**Table 2:** Individual demographic and clinical data.

Patient code	Age (years)	Sex	Etiology	TSI^a^	NIHSS^b^	GCS^c^
2020342-1	16	Female	TBI^d^	8.7	—^e^	Missing
2020342-2	38	Male	TBI	3.4	—	3
2020342-4	63	Male	TBI	3.9	—	3
2020342-5	48	Male	Ischemic stroke	5.5	18	—
2020342-6	19	Female	Hemorrhagic stroke	4.6	12	—
2020342-7	40	Male	TBI	4.2	—	Missing
2020342-8	41	Male	Hemorrhagic stroke	5.0	2	—
2020342-9	20	Male	TBI	5.3	—	4
2020342-10	19	Female	TBI	5.0	—	3
2020342-11	39	Female	Ischemic stroke	3.5	20	—
2020342-12	32	Female	Hemorrhagic stroke	3.9	2	—
2020342-13	38	Male	Brain tumor	3.1	—	—
2020342-14	25	Male	Ischemic stroke	3.3	7	—
2020342-15	58	Male	Ischemic stroke	6.5	12	—
2020342-16	51	Female	Ischemic stroke	5.5	12	—
2020342-17	29	Female	Hemorrhagic stroke	4.3	Missing	—
2020342-18	50	Female	Ischemic stroke	3.3	14	—
2020342-19	34	Male	TBI	5.4	—	Missing
2020342-20	58	Female	Hemorrhagic stroke	6.9	12	—
2020342-21	47	Male	Ischemic stroke	2.8	5	—

^a^TSI: time since injury (months).

^b^NIHSS: National Institute of Health Stroke Score.

^c^GCS: Glasgow Coma Scale.

^d^TBI: traumatic brain injury.

^e^Not available.

### Evaluation of Outcome Measures

We divided participants into separate groups based on etiology (TBI and stroke) and age (young: 16-39 years and adult: 40-63 years). We used appropriate measures of central tendency and variability, such as means and SDs (Table 3). According to each etiology and age subgroup comparison, all of them achieved more than 36 points in the UQ score, very close to the maximum of 40 points. Participant subgroups achieved more than 8 points out of 12 for being experienced in using new technologies such as personal computers, smartphones, and the internet. Regarding the manifestation of motion side effects, none of the groups achieved a minimum of 10 points on the SSQ score, indicating the absence of negative symptoms. A difference of 7.4 minutes was observed when comparing the intervention duration time between the TBI and stroke subgroups. Thus, participants with stroke scored 1 point higher in the TFQ score and completed 1 more task than participants with TBI.

**Figure 5:**
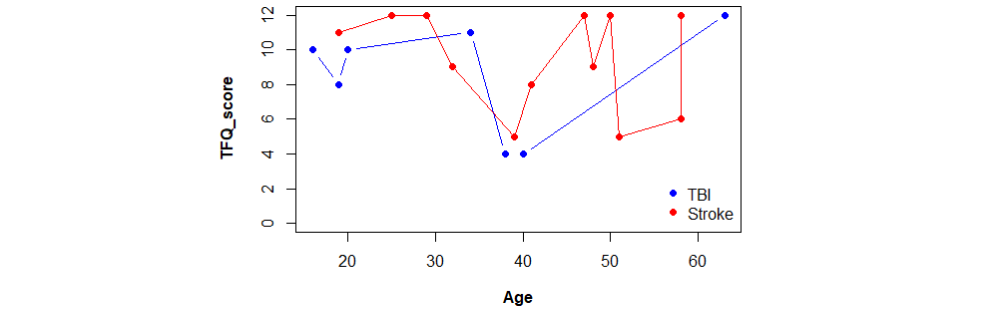
Comparison plot between TFQ scores obtained by etiology and distributed by age. TBI: traumatic brain injury; TFQ: Technology Familiarity Questionnaire.

The scores obtained by the participants in the TFQ questionnaire were compared depending on age and separated by etiology, excluding the patient with brain tumor (Figure 5). Most participants reported an acceptable level of the use of new technologies, but 5 achieved scores below half the maximum. The 2 lowest scores, 4/12, were obtained by patients with TBI. One participant, a 38-year-old male with a Glasgow Coma Scale score of 3, obtained the lowest score of 4/12. Another participant, a 40-year-old male with no available severity data, also scored 4/12. The next lowest score of 5/12 was obtained by 2 patients with moderate to severe stroke. One was a 51-year-old woman with an NIHSS of 12, and the other was a 39-year-old woman with an NIHSS of 20. Finally, a score of 6/12 was obtained by a 58-year-old male patient diagnosed with moderate stroke (NIHSS of 12). It is important to highlight that age-matched participants, even older, reported an acceptable use of new technologies.

**Table 3:** Descriptive statistics of age and TSI^a^, results of the UQ^b^, TFQ^c^, SSQ^d^, intervention duration, and number of tasks realized.

Statistic	TBI^e^ (n=7), mean (SD)	Stroke (n=12), mean (SD)	Young (n=11), mean (SD)	Adult (n=9), mean (SD)
Age	32.9 (16.5)	41.4 (12.8)	28.1 (8.7)	50.7 (7.8)
TSI	5.1 (1.7)	4.6 (1.3)	4.6 (1.6)	4.8 (1.4)
UQ	37.4 (1.7)	36.8 (3.1)	36.5 (3)	37.6 (1.9)
TFQ	8.4 (3.3)	9.4 (2.8)	9.5 (2.8)	8.9 (3.3)
SSQ	0.9 (1.9)	1.4 (2.3)	1.5 (2.4)	1 (1.6)
Duration	20.9 (3.8)	28.3 (2.8)	26.2 (3.9)	25 (5.7)
N_tasks	4.4 (1)	5.4 (0.8)	5.4 (0.9)	4.8 (1)

^a^TSI: time since injury.

^b^UQ: Usability Questionnaire.

^c^TFQ: Technology Familiarity Questionnaire.

^d^SSQ: Simulator Sickness Questionnaire.

^e^TBI: traumatic brain injury.

**Figure 6:**
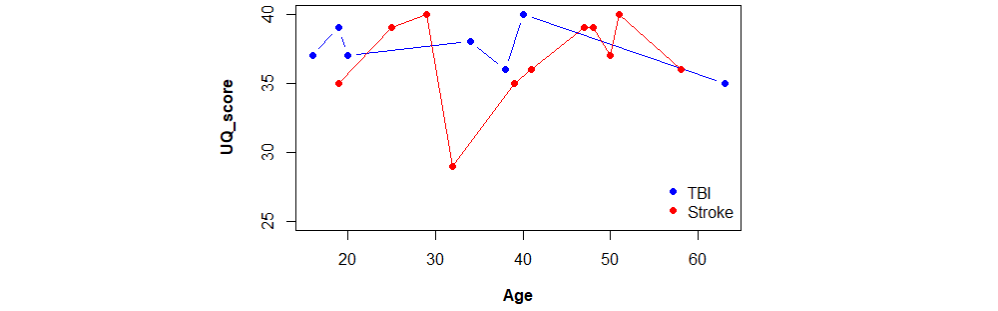
Comparison plot between UQ scores obtained by etiology and distributed by age. TBI: traumatic brain injury; UQ: Usability Questionnaire.

The mean tech familiarity score for patients with stroke (9.4) was slightly higher compared with that for patients with TBI (8.4), but this did not affect the usability scores. Overall, all participants achieved high usability scores, equal to or over 35/40, except for 1 patient, a 32-year-old woman diagnosed with a minor stroke (NIHSS of 2), who scored 29/40 points for the usability of the VR intervention (Figure 6). This could be because the patient consistently rated all questions with a 4/5, instead of assigning lower scores to some items. Additionally, she appeared indifferent regarding the occurrence of errors, as evidenced by consistently assigning a score of 3/5.

When comparing the spatial distribution of the stroke and TBI subgroups based on age, no substantial differences were observed regarding usability, by either age or etiology.

**Figure 7:**
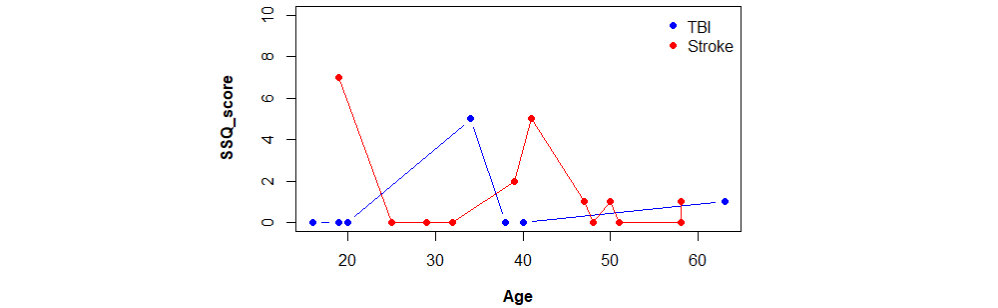
Comparison plot between SSQ scores obtained by etiology and distributed by age. SSQ: Simulator Sickness Questionnaire; TBI: traumatic brain injury.

Similarly, in terms of simulator sickness, neither the 2 etiology groups nor the patient with a brain tumor (SSQ score=2) exhibited any substantial differences in the presence of symptoms, regardless of age (Figure 7). The upper limit of the y-axis, as shown in Figure 7, has been truncated at 10. This range ensures safety by indicating the absence of simulator sickness. None of the patients obtained a score greater than this threshold.

**Figure 8:**
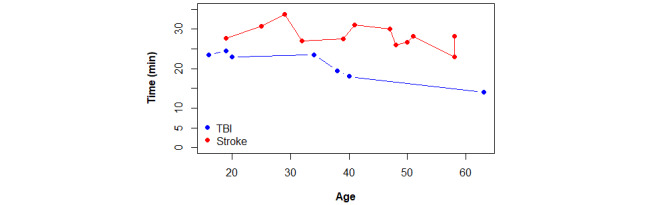
Comparison plot between intervention duration time differentiation by etiology and age.

Another aspect under examination is the duration of the VR-based intervention while wearing the headset. Following the time needed for patients to understand the intervention, fit and set up the equipment, and complete questionnaires, all participants were allotted approximately 30 minutes to engage in a series of immersive serious games. The subgroup of patients with stroke appeared to tolerate longer interventions wearing the headset compared with patients with TBI because, on average, the stroke subgroup performed more tasks. Additionally, Figure 8 illustrates a decreasing trend in the duration of VR interventions with older ages for patients with TBI.

The sample size of participants with TBI was small, but several factors may contribute to explaining these differences in time exposure. First, 2 participants with TBI completed a set of 5 tasks more quickly than those with stroke, possibly because they were on average 10 years younger (see Table S2 in Multimedia Appendix 4). According to the literature, younger age correlates with faster reaction times [60]. By contrast, an adult participant with TBI (code 2020342-19) reported feelings of dizziness and pixelated vision (see Table S2 in Multimedia Appendix 3). He stopped mid-intervention to remove the VR glasses and rest for a couple of minutes. Additionally, the oldest patient in the entire sample was from the TBI subgroup and was the one who requested to finish early, completing only 3 tasks. These occurrences contributed to a shorter intervention time for the TBI subgroup.

**Figure 9:**
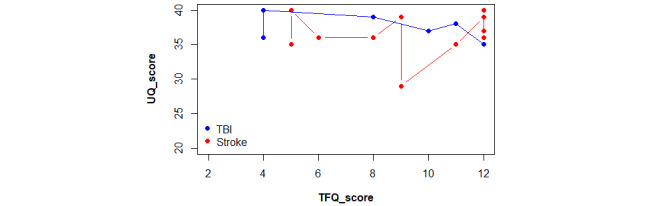
Comparison plot between UQ and TFQ scores, separated by etiology. TBI: traumatic brain injury; TFQ: Technology Familiarity Questionnaire; UQ: Usability Questionnaire.

Based on this rationale and observing the result of the comparison between UQ scores and TFQ scores (Figure 9), the co-designed and developed VR-based cognitive rehabilitation support tool appears to be feasible when applied in the hospital setting and with patients with ABI. It demonstrates high usability regardless of age, the origin of the lesion, and familiarity with new technologies.

**Figure 10:**
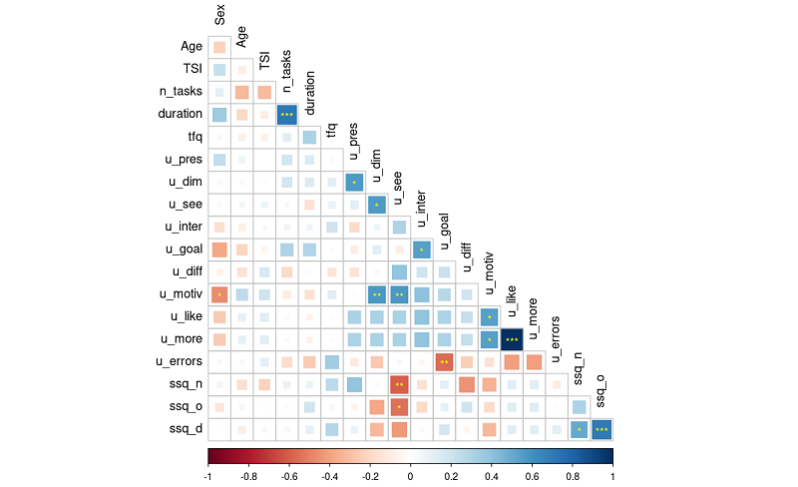
Correlations between sample’s demographic characteristics, number of tasks completed, session duration time, technology familiarity, usability categories, and SSQ. *P<.05, **P<.01, and ***P<.001. SSQ: Simulator Sickness Questionnaire; TFQ: Technology Familiarity Questionnaire; TSI: time since injury.

We also investigated the correlations among Simulator Sickness subscale symptoms, usability categories, tech familiarity scores, age, TSI, number of tasks performed, and time wearing the VR headset (Figure 10). The intensity of the square’s color is directly proportional to the strength of the correlations between variables. Positive correlations are labeled with cool colors, whereas negatives are warm. Significant correlations are indicated with asterisks. The exact *P* values are presented in Table S1 in Multimedia Appendix 5.

There were significant, strong correlations between some variables included in the analysis. The data extracted from the session performance were closely related, and therefore, the intervention duration positively correlated with the number of tasks performed (*r*=0.72, *P*<.001), as expected. Regarding usability categories, the dimensions matching (u_dim) correlated with the sense of presence (u_pres: *r*=0.56, *P*=.01) and with the ability to see and differentiate objects (u_see: *r*=0.56, *P*=.01). The task goal-specificity (u_goal) correlated positively with the ability to interact with the environment (u_inter: *r*=0.55, *P*=.01). The motivation prompted by the intervention (u_motiv) correlated with the dimensions matching (u_dim: *r*=0.57, *P*=.008) and with the ease in seeing and differentiating objects (u_see: *r*=0.57, *P*=.008). Additionally, motivation correlated with sex, considering that 0 corresponds to the male sex and 1 to the female sex. As the sign of the correlation is negative, a strong correlation between male sex and motivation was observed (*r*=–0.46, *P*=.04) [61]. Furthermore, the liking of VR interventions (u_like) and the desire to conduct more VR in rehabilitation programs (u_more) were correlated (*r*=0.56, *P*<.001), and both were also correlated with the motivation experienced (u_motiv) with similar results (*r*=0.55, *P*=.01). The presence of errors that some participants had reported correlated negatively with the ability to understand and achieve the goal of the task (*r*=–0.56, *P*=.009).

Finally, concerning the SSQ symptoms analyzed, a strong correlation between disorientation (ssq_d) and oculomotor problems (ssq_o) was observed (*r*=0.71, *P*<.001). The disorientation sickness symptoms also correlated with the nausea sickness symptoms (ssq_n: *r*=0.50, *P*=.02). Additionally, the nausea symptoms and oculomotor problems negatively correlated with the ease in seeing and differentiating objects (*r*=–0.60, *P*=.005 and *r*=–0.54, *P*=.01, respectively).

## Discussion

### Support Tool Developed

The VR-based support tool proposed in this study comprised a high-end new-generation commercial device, namely, the HTC VIVE Pro Eye, along with a series of custom tasks designed to rehabilitate cognitive functions (eg, attention, memory, and executive functions) in patients with ABI. These patients were undergoing neurorehabilitation treatment at a health care center. The overall satisfaction percentage achieved by the sample of 20 patients, considering the usability score and the evaluation of side effects, was 89.8% (431/480; 37/40 usability points, subtracting 1.3 from 48 SSQ points). The system was developed following recent recommendations [27,28] combined with our approach to how VR applications should be designed for clinical trials (Textbox 1). The results obtained from this study may contribute to filling the gap in the literature related to the lack of studies that follow a methodological process of best practices to integrate VR technology as a neurorehabilitation support tool for patients with ABI in the daily practice of real hospital settings [24,25,62].

**Stepwise summarized approach to achieve a virtual reality–based neurorehabilitation support tool for inpatients with acquired brain injury.**

1. **Identification of virtual reality (VR)–based intervention needs and barriers for patients with acquired brain injury (ABI)**
  - A multidisciplinary meeting involving health professionals and researchers identified the need for a VR-based neurorehabilitation support tool for patients with ABI.
  - Difficulties and barriers were identified, and possible solutions were proposed, in collaboration with technologists and VR experts.
  - The first approach to VR support tool features and treatment interventions was defined.
2. **Selection and placement of technological device**
  - A high-end new-generation immersive system was selected and placed within the hospital setting.
  - Device testing with available off-the-shelf VR games was conducted with clinical professionals and end users.
3. **Co-design of VR-based neurorehabilitation support tool**
  - Physical medicine and rehabilitation physicians, neuropsychologists, therapists, and nurses targeted the patient population and desired intervention.
  - Ideas for new VR experiences were generated, addressing different cognitive or sensorimotor functions.
  - Researchers and developers created the first sketches based on technology capabilities and current knowledge.
  - Immersive serious games, rehabilitative principles, game mechanics, interactions, sound and effects, graphic environment, and measurable data, among other features were discussed.
4. **Prototyping**
  - Developers built prototypes, which were tested and redesigned by co-designers until desired behavior and appearance, maximum safety, easy, and a quick set up were guaranteed.
  - Input and output variables with configurable thresholds were determined.
  - Approaches to minimize cybersickness symptoms, simplified interactions, and multisensory feedback incorporation were used.
  - Use cases were performed involving treatment providers and end users.
  - A set of immersive serious games, including neurorehabilitation principles, was achieved.
5. **Usability and feasibility study**
  - A study protocol was defined, including participant characteristics (inclusion/exclusion criteria), intervention, and outcome measures.
  - Target patients were recruited.
  - Multiple proof-of-concept studies were conducted.
  - Demographics, clinical data, in-game measures, and structured questionnaire responses were collected.
  - Statistical analyses were performed, and results were discussed.
6. **Basis for future research**
  - Requirements of the VR support tool for patients with ABI were elicited.
  - The foundation was established for future large study designs to determine the efficacy of VR interventions.

### Principal Findings

Our systematic approach to developing a VR-based neurorehabilitation support tool for inpatients with ABI has resulted in a set of 7 cognitive tasks specifically designed to address the needs of this population. The sample of 20 patients, with a mean TSI of 4.7 (SD 1.5) months, volunteered to participate in assessing the usability and feasibility of the proposed intervention. Participants completed an average of 5 tasks during a single VR session lasting approximately 25 minutes. The set of cognitive tasks was well-received by participants, irrespective of etiology, age, or tech familiarity.

What was significant in this study regarding the achievement of the VR tool and subsequent intervention was the step-by-step approach with the participation of stakeholders throughout the entire process, from design to prototyping, and usability and feasibility assessment. By applying this methodology, we have demonstrated the potential of integrating VR into clinical practice. This supports recent literature findings that also describe detailed customized VR rehabilitation tools and have conducted large-quality studies obtaining promising results [36,39-41,63,64]. All participants from the multiple proof-of-concept study completed the session without experiencing adverse effects or encountering major issues. By targeting multiple areas of functionality, patients can benefit from a more comprehensive and personalized rehabilitation program, which can promote neuroplasticity and potentially improve overall functional outcomes [14,30].

The results demonstrated that when patients enjoyed the tasks, their motivation increased; eventually, they expressed a desire to participate in more VR sessions as part of their rehabilitation programs. This engagement was correlated with a high sense of presence, the ability to perceive and differentiate objects within the virtual environment, and a perception of real-world scale [32,65]. The study also demonstrated that when interactions are customized to fit the abilities of individual patients, their performance in completing the required tasks improves, resulting in greater clarity and specificity of the intended goal [20]. However, when tasks contain errors, it becomes more challenging for patients to understand and achieve the objectives. For example, one patient (code 2020342-2) reported difficulty in hitting the mark when shooting stimuli. This issue will be addressed by incorporating a laser pointer for future studies.

When evaluating cybersickness effects, a strong correlation was observed between patients reporting disorientation and the presence of oculomotor problems and nausea symptoms. This indicates that an increase in one of these symptoms tends to coincide with an increase in the others [49]. Furthermore, when patients reported experiencing nausea symptoms or oculomotor problems, their ability to see and differentiate objects within the scene decreased. Despite the correlations found, the overall average score for the SSQ does not exceed 1.3 points, with a maximum of 1.5 points in the subgroup of young patients (up to 39 years old). This score is still far from the threshold of 10 points, beyond which cybersickness symptoms can cause problems.

There is a demographic correlation between sex and motivation, indicating that men found the VR session more motivating than women [61]. No significant correlation was observed with the age variable. This finding, together with the comparisons of descriptive statistics, may support the evidence that VR is a useful and viable tool for different age groups, ranging from 16 to 63 years old. However, it is important to interpret these findings with caution, as the sample size is not sufficiently large, and only 1 session has been tested, rather than a long-term intervention with a follow-up assessment.

The commercial device selected was suitable for inpatient rehabilitation, in accordance with previous studies [44,66,67]. The headset ensures comfort, improved visual quality, and exposure to graphics, along with selectable handheld controllers, a precise tracking system, and portability. Moreover, the headband and facial interface that come into contact with the patient can be replaced to reduce the risk of spreading infection among patients sharing the same device. The screen, other parts of the headset, and controllers can be disinfected using hydroalcoholic gel. Successful integration of the device within hospital settings, without hindering the use of other rehabilitative tools or treatment programs, is assured. As for the economic feasibility of acquiring the proposed system, both SteamVR and OpenVR software components are freely available for use. The Unity3D game engine provides various licensing options, including a free version. The necessary hardware comprises the following: (1) a mid-range gaming personal computer equipped with a VR-ready graphics card, priced between €1000 (US $1081) and €3000 (US $3244); (2) a high-end VR input/output device such as Valve Index or Oculus, typically priced around €1200 (US $1297); and (3) potential expenses may arise from hiring developers or subcontractors to create the virtual environments.

### Limitations

While our study offers valuable insights into the utilization of VR-based tools for cognitive rehabilitation in patients with ABI, it is important to acknowledge several limitations that warrant attention. Primarily, there exists a discrepancy in the number of tasks targeting each cognitive domain. Despite this variance, it is crucial to emphasize that the obtained results were adequate for identifying and delineating crucial aspects of feasibility and usability. Future studies assessing efficacy should encompass a balanced array of tasks targeting each cognitive domain. This approach will facilitate more comprehensive and intensive interventions, addressing the spectrum of cognitive impairments observed in patients with ABI.

In line with this, it would be compelling to broaden our intervention to encompass other realms of rehabilitation, such as upper and lower limb function, gait analysis, mirror therapy, and pain management, among others.

Another limitation is the absence of a centralized server for gathering output variables generated by each task. For future studies aiming to obtain efficacy results, ascertain which data trigger changes during the neurorehabilitation process, and develop predictive models to personalize treatments, having such a server would be invaluable.

Furthermore, certain patients’ clinical records contained missing data regarding the severity scales, potentially affecting the analysis of results. The complete tables, encompassing all collected variables including individual responses to questionnaires, are available for reference in Multimedia Appendices 3 and 4.

Finally, although the design and refinement of the VR experiences were conducted by a multidisciplinary team comprising health professionals and end users, structured questionnaires were not administered to them during this process. However, a log detailing meetings, tests conducted, the primary themes explored, alterations made, error corrections, and some feedback was prepared (see Table S1 in Multimedia Appendix 1).

### Conclusions

Based on our understanding, this study holds significance as it lays the foundation for a VR-based neurorehabilitation support tool applicable to a wide spectrum of patients with ABI within the practical context of a hospital setting. The process of requirement elicitation and iterative development was meticulously conducted in collaboration with a multidisciplinary team, aligning closely with the latest recommendations from the literature.

This study provides evidence demonstrating the utility and feasibility of VR-based treatments when tailored to meet the specific needs of a targeted patient population. It underscores the significance of collaborative intervention design involving physicians, physiotherapists, neuropsychologists, occupational therapists, nurses, researchers, technologists, and incorporating patient perspectives. The intervention ought to encompass a diverse range of immersive experiences, drawing upon neurorehabilitation principles and serious games techniques while ensuring ecological validity. By adhering to this approach, VR-based interventions hold the potential to provide valuable support in neurorehabilitation settings.

Future studies should aim to conduct rigorous research with larger sample sizes and robust study designs to offer more substantial evidence regarding the clinical value and cost-effectiveness of VR-based interventions in the neurorehabilitation of patients with ABI. For this purpose, a clinical efficacy study is already in progress. The ultimate objective is to develop a standard operating procedure that facilitates reproducibility, comparison, and generalization of findings.

## Appendix

Multimedia Appendix 1 VR-based support tool acquisition procedure. VR: virtual reality.

Multimedia Appendix 2 Setup photos.

Multimedia Appendix 3 Questionnaire models and results.

Multimedia Appendix 4 Demographic and outcome measures data of the sample.

Multimedia Appendix 5 Correlation matrix results.

## Abbreviations

ABI: acquired brain injury
HMD: head-mounted display
NIHSS: National Institute of Health Stroke Score
SSQ: Simulator Sickness Questionnaire
TBI: traumatic brain injury
TFQ: Technology Familiarity Questionnaire
TSI: time since injury
UQ: Usability Questionnaire
VR: virtual reality
