# Supplementary material for: Virtual Reality–Based Neurorehabilitation Support Tool for People With Cognitive Impairments Resulting From an Acquired Brain Injury: Usability and Feasibility Study
Source: JMIR Neurotechnol. 2024 Mar 18;3:e50538. doi: 10.2196/50538 (PMC12671297; doi:10.2196/50538)
Supplement: Multimedia Appendix 1 [file neuro_v3i1e50538_app1.pdf]

VR-based support tool Acquisition Procedure

This is a Multimedia Appendix to a full manuscript published in the J Med Internet Res. For full copyright and citation information see http://dx.doi.org/10.2196/jmir.50538

Table 1. Record of meetings and tests.

Date	Description	Participants	Feedback
August 2020	**Meeting**. VR[a] for NRHB[b].	PM&R physicians, neuropsychologists, therapists, nurses, researchers, and technologists.	VR as a support tool for NRHB: needs, benefits, and limitations.<br>Generation of new Ideas.<br>Equipment selection and space configuration.
September 2020	**Meeting**. VR-based support tool for cognitive rehabilitation.	Neuropsychologists, researchers, and technologists.	Study design definition to evaluate a VR-based support tool for cognitive rehabilitation in patients with ABI[c].
November 30, 2020	**Meeting**. VR-based support tool for cognitive rehabilitation.	Neuropsychologists, researchers, and technologists.	VR experiences definition and classification among different cognitive functions.<br>4 existing custom experiences (attentional) need to be accommodated for the new study design.
February 9, 2021	**Meeting.** VR environments for cognitive rehabilitation.	Neuropsychologists, researchers, and technologists.	Co-design of new interactive environments of immersive VR for different cognitive functions rehabilitation (1 attentional, 2 memory, 2 executive functions).
March – April – May 2021	**Prototyping.** Preliminary VR cognitive tasks tests.	Researchers, and technologists.	Revision of 5 attentional, 1 memory, and 2 executive functions developed cognitive co-designed tasks in terms of algorithms approach, interactions, system performance, and behavior.
June 16, 2021	**Prototyping.** VR cognitive tasks test.	Neuropsychologists, researchers, and technologists.	Assessment and redesign of developed preliminary tasks. Memory task discarded.<br>Graphics and 3D models definition.<br>Co-design of new VR memory task.
September – October 2021	**Prototyping.** VR cognitive tasks tests.	Researchers, and technologists.	Follow-up of 5 attentional, 1 memory, and 2 executive functions developed cognitive co-designed tasks.

November 2, 2021	**Prototyping.** VR cognitive tasks.	Neuropsychologists, researchers, and technologists.	Assessment and redesign.<br>Difficulty-dependent variables adjustment. Feedback incorporation (visual and haptic). Refine interactions. Refine graphics.
December 1, 2021	**Prototyping.** VR cognitive tasks.	Neuropsychologists, researchers, and technologists.	Assessment and redesign.<br>Adaptative algorithms incorporation. Adjusting in-game variables. More feedback incorporation (visual). Refine graphic details.
February 2, 2022	**Meeting.** Study protocol for cognitive rehabilitation using VR support tools.	Neuropsychologists, researchers, and technologists.	VR cognitive tasks preparation for a feasibility and usability study.
March 1, 2022	**Prototyping.** VR cognitive tasks.	Neuropsychologists, researchers, and technologists.	Assessment and last details fixed. 1 attentional task was discarded for the study protocol.<br>A set of 7 cognitive tasks ready to test with health professionals and end-users.
7-11 March 2022	**Proof of Concept**. VR cognitive tasks with treatment providers and end-users.	Patients with ABI, neuropsychologists, researchers, and technologists.	"*The degree of immersion is excellent. Some task gets long. The glasses are comfortable and the tasks intuitive. The difficulty is also well-tuned.*" Several proposals have been collected regarding playability, visual clues to guide neglected patients, and events to increase the number of visual stimuli at the time, to provide additional difficulty levels.
March 23, 2022	**Meeting.** VR-based support tool for cognitive rehabilitation.	Neuropsychologists, researchers, and technologists.	Send the proposal for a protocol to carry out the study entitled "Application of Virtual Reality to cognitive treatment in Patients with Acquired Brain Injury" to the ethics committee.
March 30, 2022	**Proofs of Concept.** VR tool for NRHB.	Patients with ABI, treatment providers, researchers, and technologists.	Tasks can be performed in both standing and sitting positions. With the first one, the treatment provider will support the patient to prevent falls.<br>"Training on best practices to set up the system is needed. The steps to turn on the system,

			prepare the session, and put the headset and controllers to the patient."<br>"It may be difficult to aim the faster meteorites." A proposal to add a laser pointer is collected.<br>"It would be nice to use tracking devices instead of remotes for tasks that don't require pushing buttons."
April 29, 2022	**Meeting.** VR-based support tool for cognitive rehabilitation.	Neuropsychologists, researchers, and technologists.	Protocol approved. Informed consent and questionnaires ready.
May 9-13, 2022	**VR placement.** VR setup for clinical trials.	Neuropsychologists, technologists.	Room configuration for VR sessions within the hospital setting.
May 18, 2022	**Proofs of Concept.** VR tool for cognitive rehabilitation.	Neuropsychologists, researchers, and technologists.	"*The VR session was good, I really liked it. Tasks become shorter when played than when supervised from outside*." Some proposals have been collected regarding methods to adapt the movement capacity needed to intercept stimuli for the more impeded patients, and events to provide additional difficulty levels.
20-24 June 2022	**Prototyping.** VR cognitive tasks.	Neuropsychologists, researchers, and technologists.	Final assessment of the latest modifications derived from the concept tests.<br>The system is ready to start the usability and feasibility study.
27-30 June 2022	**U&F[d] Proofs of Concept.** VR tool for cognitive rehabilitation.	Patients, treatment providers, researchers, and technologists.	Demographics, clinical data, in-game measures, and questionnaire collection.
July 2022	**U&F Proofs of Concept.** VR tool for cognitive rehabilitation.	Patients, treatment providers, researchers, and technologists.	Demographics, clinical data, in-game measures, and questionnaire collection.
8-12 August 2022	**U&F Proof of Concept.** VR tool for cognitive rehabilitation.	Patients, treatment providers, researchers, and technologists.	Demographics, clinical data, in-game measures, and questionnaire collection.

[a]Virtual Reality

[b]Neurorehabilitació

[c]Acquired Brain Injury

[d]Usability and feasibility

Table 2. Details of the multidisciplinary team involved in the VR-based support tool acquisition procedure (co-design and prototyping).

**Profile[a]**	**N (N researchers)**	**Main details**
Physiotherapist	9 (2)	Research team members identified needs and proposed approaches to obtain new VR-based tools. Technologists developed initial prototypes. Health professionals from the research team tested the initial prototypes. Corresponding changes were applied. Health professionals not from the research team were asked to test advanced prototypes. As feedback was collected, they learned to manipulate the tool. Prototypes were tested and redesigned until desired behavior, maximum safety, and easy and quick set-up were guaranteed.
Occupational therapist	3 (0)	
Neuropsychologist	6 (3)	
Researcher	9 (9)	
Technologist	2 (2)	
Patient with ABI	9 (0)	Patients from childhood to youth to advanced age, from both sexes and etiology of Stroke, TBI, or Brain Tumour, were asked to test the most advanced prototypes being developed. They were undergoing functional training in the rehabilitation gym, and they understood basic instructions, had enough mobility to manipulate a controller with at least one hand, did not suffer from epilepsy or vertigo, and could be fitted with glasses. Positive feedback was appreciated, and valuable comments and observations were collected for the final VR-based tool acquisition.

[a] All profiles correspond to individuals working or being patients from Institut Guttmann.
