# Supplementary material for: Virtual Reality–Based Neurorehabilitation Support Tool for People With Cognitive Impairments Resulting From an Acquired Brain Injury: Usability and Feasibility Study
Source: JMIR Neurotechnol. 2024 Mar 18;3:e50538. doi: 10.2196/50538 (PMC12671297; doi:10.2196/50538)
Supplement: Multimedia Appendix 2 [file neuro_v3i1e50538_app2.pdf]

Setup Photos

This is a Multimedia Appendix to a full manuscript published in the J Med Internet Res. For full copyright and citation information see http://dx.doi.org/10.2196/jmir.50538

Figure 1. Photos of the setup during Usability and Feasibility Proofs of Concept sessions with patients with Acquired Brain Injury.

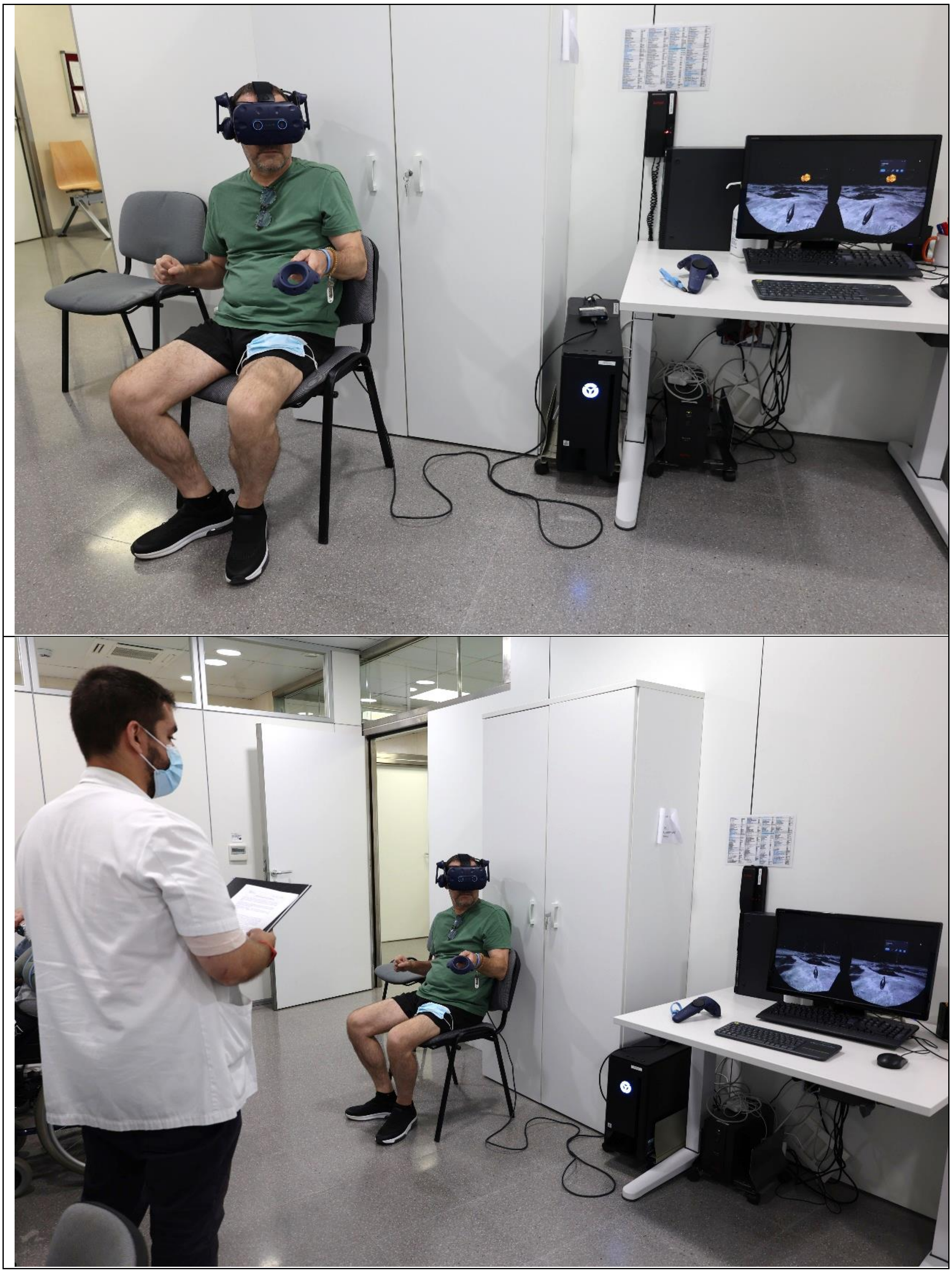

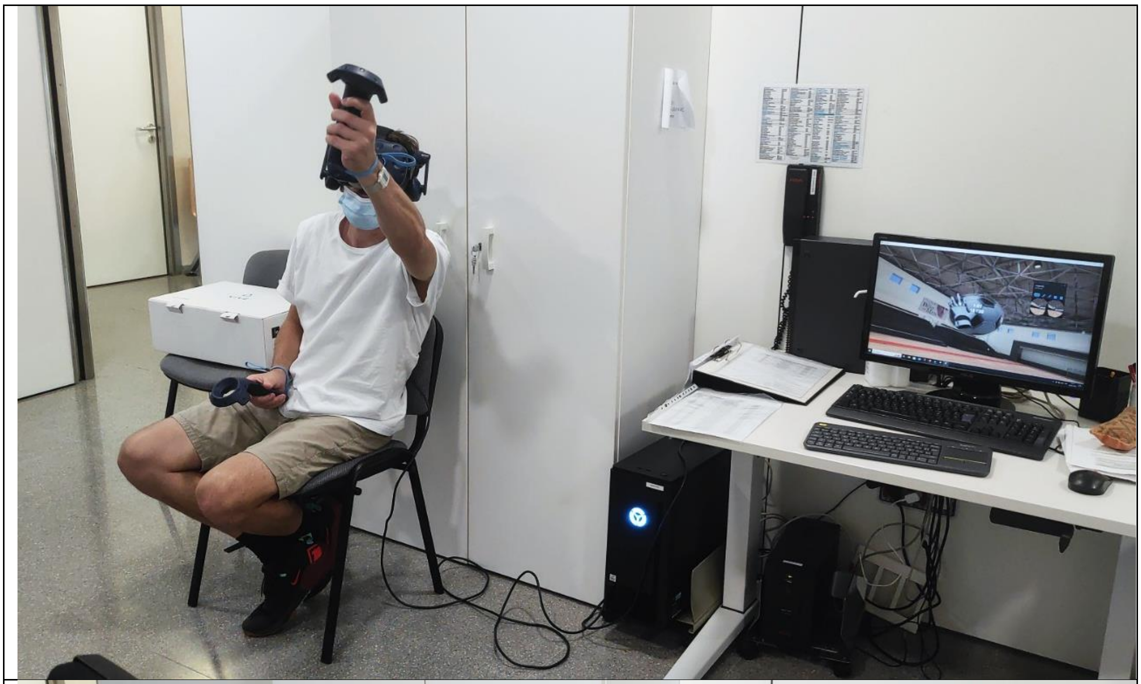

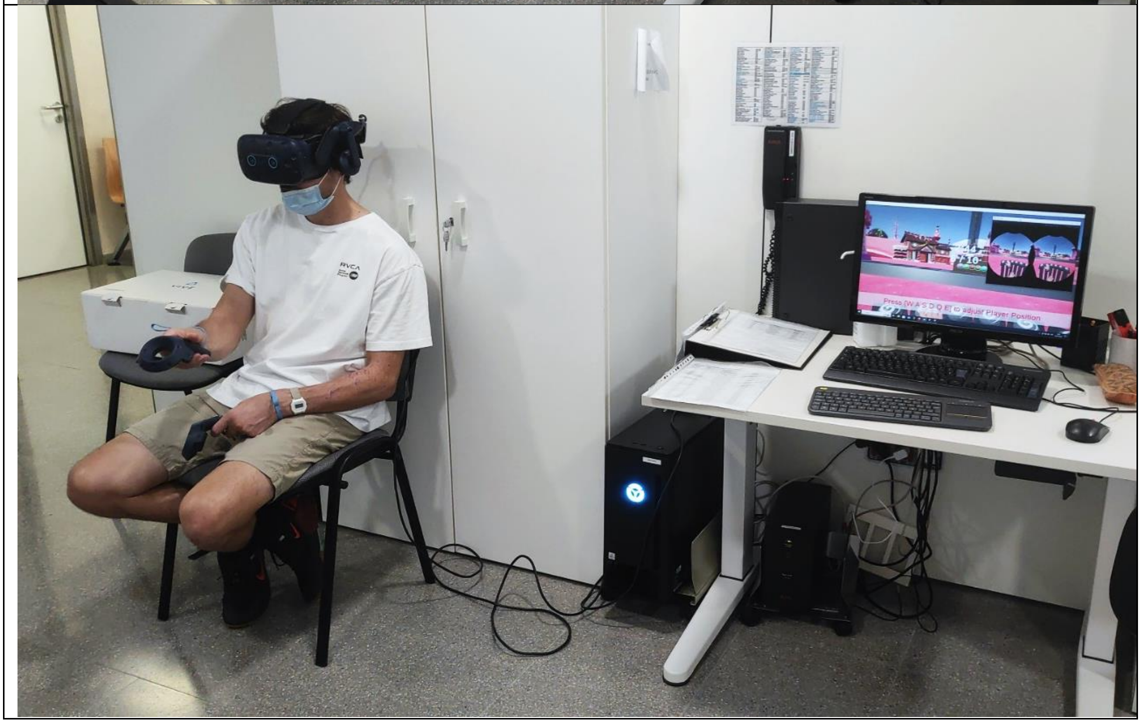
RVCA
