# Supplementary material for: Virtual Reality–Based Neurorehabilitation Support Tool for People With Cognitive Impairments Resulting From an Acquired Brain Injury: Usability and Feasibility Study
Source: JMIR Neurotechnol. 2024 Mar 18;3:e50538. doi: 10.2196/50538 (PMC12671297; doi:10.2196/50538)
Supplement: Multimedia Appendix 3 [file neuro_v3i1e50538_app3.pdf]

Questionnaire Models and Results

This is a Multimedia Appendix to a full manuscript published in the J Med Internet Res. For full copyright and citation information see http://dx.doi.org/10.2196/jmir.50538

Table 1. Structured Questionnaires delivered to participants: Usability, Tech Familiarity, and Simulator Sickness.

**Questionnaire, number**	**Category**	**Question**
**Usability**		
1	Sense of presence	When I put on the VR glasses, I feel like I'm in the virtual world I see
2	Dimensions matching	The size of the objects and the scene is appropriate. I don't feel like I'm too tall or too short in the virtual world
3	See	It is easy for me to see and differentiate the objects that appear in the virtual world
4	Interactivity	I find it easy to interact with the environment and manipulate virtual objects
5	Task Specificity	I am clear about the purpose of the task and what I have to do
6	Task Difficulty	The difficulty of the task is adapted to my abilities. I am neither bored nor stressed
7	Motivation	I find VR activities motivating
8	Like	In general, I like how the activities are presented (virtual environments, tasks to be done) and I liked the session
9	More VR	I would be pleased if VR activities were in the daily rehabilitation programs
10	Errors	There are errors in the program that must be fixed
	Open-ended question	What errors?
**Technology Familiarity**		
1	PC	How often do you use a computer at work and/or at home?
2	Smartphone	How often do you use a smartphone at work and/or at home?
3	Internet	How often do you use internet at work and/or at home?
**SSQ**		
1	General discomfort (N[a], O[b])	When I do VR activities, I feel a general discomfort

2	Fatigue (O)	When I do VR activities, I feel fatigue
3	Headache (O)	When I do VR activities, gives me a headache
4	Eye strain (O)	Doing VR activities causes eye strain
5	Difficulty in eye focusing (O, D[c])	I have difficulty in eye focusing when doing VR activities
6	Increased salivation (N)	I notice an increase in salivation when doing VR activities
7	Sweating (N)	I notice an increase in sweat when doing VR activities
8	Nausea (N, D)	Doing VR activities makes me nauseous
9	Difficulty in concentration (N, O)	I have difficulty concentrating when doing VR activities
10	Fullness of head (D)	I have a feeling of fullness in my head when doing VR activities
11	Blurred vision (O, D)	When I do VR activities, my vision becomes blurred
12	Dizzy – eyes open (D)	I feel dizzy when doing VR activities with my eyes open
13	Dizzy – eyes close (D)	I feel dizzy when doing VR activities with my eyes close
14	Vertigo (D)	When I do VR activities, I feel vertigo
15	Stomach Awareness (N)	When I do VR activities, I feel sick to my stomach
16	Burping (N)	Doing VR activities makes me burp

[a]Nausea subscale symptoms included.
[b]Oculomotor problems subscale symptoms included.
[a]Disorientation subscale symptoms included.

Table 2. Results of Usability Questionnaire: every single question, final score, and reported comments.

Patient ID	U_Pres	U_Dim	U_See	U_Inter	U_Goal	U_Diff	U_Motiv	U_Like
2020342-1	4	4	4	4	3	4	4	4
2020342-2	4	4	4	3	3	3	4	4
2020342-4	3	3	4	4	3	4	4	4
2020342-5	4	4	4	4	4	4	4	4
2020342-6	4	3	3	4	4	3	3	4
2020342-7	4	4	4	4	4	4	4	4
2020342-8	3	3	3	4	4	4	4	4
2020342-9	3	3	4	4	4	4	4	4
2020342-10	3	4	4	4	4	4	4	4
2020342-11	4	3	3	3	3	4	3	4
2020342-12	3	3	3	3	3	3	3	3
2020342-13	4	4	3	4	4	2	4	4
2020342-14	3	4	4	4	4	4	4	4
2020342-15	3	3	3	4	4	3	4	4

2020342-16	4	4	4	4	4	4	4	4
2020342-17	4	4	4	4	4	4	4	4
2020342-18	4	4	4	4	3	3	4	4
2020342-19	4	4	3	3	4	4	4	4
2020342-20	4	4	4	3	3	3	4	4
2020342-21	4	4	4	4	4	4	4	4

Continuation:

Patient ID	U_More	U_Errors	U_TS	Errors comments
2020342-1	4	2	37	-
2020342-2	4	3	36	When you shoot, it is difficult to hit the mark
2020342-4	4	2	35	-
2020342-5	4	3	39	-
2020342-6	4	3	35	In the butterfly task, most of the butterflies are far away from my hands
2020342-7	4	4	40	-
2020342-8	4	3	36	-
2020342-9	4	3	37	-
2020342-10	4	4	39	-
2020342-11	4	4	35	-
2020342-12	3	2	29	-
2020342-13	4	3	36	-
2020342-14	4	4	39	-
2020342-15	4	4	36	-
2020342-16	4	4	40	-
2020342-17	4	4	40	-
2020342-18	4	3	37	-
2020342-19	4	4	38	The vision in some tasks was pixelated and caused dizziness. There is a that you can hit between controllers.
2020342-20	4	3	36	-
2020342-21	4	3	39	-

Table 3. Results of Technology Familiarity Questionnaire: every single question and final score.

Patient ID	TF_PC	TF_Phone	TF_Internet	TF_TotalScore
2020342-1	2	4	4	10
2020342-2	1	2	1	4
2020342-4	4	4	4	12
2020342-5	3	3	3	9
2020342-6	3	4	4	11
2020342-7	1	2	1	4
2020342-8	3	3	2	8
2020342-9	3	4	3	10
2020342-10	1	3	4	8
2020342-11	1	3	1	5

2020342-12	3	3	3	9
2020342-13	4	4	4	12
2020342-14	4	4	4	12
2020342-15	2	2	2	6
2020342-16	2	2	1	5
2020342-17	4	4	4	12
2020342-18	4	4	4	12
2020342-19	4	4	3	11
2020342-20	4	4	4	12
2020342-21	4	4	4	12

Table 4. Results of SSQ: every single question.

**Symptom**	**2020342-1**	**2020342-2**	**2020342-4**	**2020342-5**	**2020342-6**	**2020342-7**	**2020342-8**	**2020342-9**
General discomfort	0	0	0	0	0	0	0	0
Fatigue	0	0	0	0	0	0	1	0
Headache	0	0	0	0	1	0	0	0
Eyestrain	0	0	0	0	0	0	2	0
Difficulty focusing	0	0	1	0	1	0	1	0
Increased salivation	0	0	0	0	0	0	0	0
Sweating	0	0	0	0	2	0	0	0
Nausea	0	0	0	0	0	0	0	0
Difficulty concentration	0	0	0	0	0	0	0	0
Fullness of head	0	0	0	0	1	0	0	0
Blurred vision	0	0	0	0	0	0	1	0
Dizzy (eyes open)	0	0	0	0	1	0	0	0
Dizzy (eyes closed)	0	0	0	0	0	0	0	0
Vertigo	0	0	0	0	1	0	0	0
Stomach awareness	0	0	0	0	0	0	0	0
Burping	0	0	0	0	0	0	0	0

Continuation:

**Symptom**	**2020342-10**	**2020342-11**	**2020342-12**	**2020342-13**	**2020342-14**	**2020342-15**
General discomfort	0	1	0	0	0	0
Fatigue	0	0	0	0	0	0
Headache	0	0	0	0	0	0
Eyestrain	0	1	0	0	0	0
Difficulty focusing	0	0	0	0	0	0

Increased salivation	0	0	0	1	0	0
Sweating	0	0	0	1	0	0
Nausea	0	0	0	0	0	0
Difficulty concentration	0	0	0	0	0	0
Fullness of head	0	0	0	0	0	0
Blurred vision	0	0	0	0	0	0
Dizzy (eyes open)	0	0	0	0	0	0
Dizzy (eyes closed)	0	0	0	0	0	0
Vertigo	0	0	0	0	0	0
Stomach awareness	0	0	0	0	0	0
Burping	0	0	0	0	0	0

Table 5. Results of SSQ: total score and grouped symptoms score.

**Patient ID**	**SSQ_TS**	**SSQ_N**	**SSQ_O**	**SSQ_D**
2020342-1	0	0	0	0
2020342-2	0	0	0	0
2020342-4	1	0	1	1
2020342-5	0	0	0	0
2020342-6	7	2	2	4
2020342-7	0	0	0	0
2020342-8	5	0	5	2
2020342-9	0	0	0	0
2020342-10	0	0	0	0
2020342-11	2	1	2	0
2020342-12	0	0	0	0
2020342-13	2	2	0	0
2020342-14	0	0	0	0
2020342-15	0	0	0	0
2020342-16	0	0	0	0
2020342-17	0	0	0	0
2020342-18	1	1	0	0
2020342-19	5	2	3	2
2020342-20	1	0	1	1
2020342-21	1	0	1	1
